# Isolation and characterization of a new fructophilic *Lactobacillus plantarum* FPL strain from honeydew

**DOI:** 10.1007/s13213-018-1350-2

**Published:** 2018-06-01

**Authors:** Klaudia Gustaw, Magdalena Michalak, Magdalena Polak-Berecka, Adam Waśko

**Affiliations:** Faculty of Food Science and Biotechnology, Department of Biotechnology, Microbiology and Human Nutrition, University of Life Sciences in Lublin, Skromna 8, 20-704 Lublin, Poland

**Keywords:** *Lactobacillus plantarum*, *Coccus hesperidum* L., Fructophilic lactic acid bacteria, Honeydew, Isolation, Syntrophic bacteria

## Abstract

In the present study, a *Lactobacillus plantarum* FPL strain exhibiting fructophilic behavior has been isolated for the first time from honeydew. It is a probably syntrophic bacterium inhabiting the gastrointestinal tract of *Coccus hesperidum* L. and taking part in sugar metabolism. The promising growth characteristics and biochemical properties of *Lb. plantarum* FPL indicate that this may be a facultatively fructophilic species, whose properties are not associated with the loss of the alcohol/acetaldehyde dehydrogenase gene. The article attempts to classify the peculiar behavior of this strain by means of tests that are characteristic for FLAB as well as through a classic identification approach. In this study, we used a reference strain *Lb. plantarum* NRRL B-4496, which showed no fructophilic properties. With the FLAB group, the new strain shares the habit, such as a fructose-rich environment, the preference of this sugar for growth, and similar growth curves. However, it exceeds FLAB in terms of osmotolerance to high sugar content. The fructophilic *Lb. plantarum* FPL strain can proliferate and grow on a medium wherein the sugar concentration is 45 and 50% (*w*/*v*). Our findings indicate that honeydew can be a promising source of new fructophilic lactic acid bacteria.

**Electronic supplementary material:**

The online version of this article (10.1007/s13213-018-1350-2) contains supplementary material, which is available to authorized users.

## Introduction

Lactic acid bacteria (LAB) are an example of organisms that evolve depending on the environment in which they live (Douglas et al. 2015). Lactic acid bacteria are generally auxotrophic for some compounds; they are quite demanding nutritionally and limited in their environmental tolerances (Christiansen et al. 2008; Gomaa and Rushdy 2014). This description contrasts with the biodiversity known today and the ability to tolerate extraordinary habitats, given the progressive knowledge of LAB genomes (Azcarate-Peril and Klaenhammer 2010; Franz and Holzapfel 2011). The ongoing reduction of the genome called “reductive evolution” (van de Guchte et al. 2006) together with acquisition or overexpression of genes (van de Guchte et al. 2006; Callanan et al. 2008; Azcarate-Peril et al. 2009) may explain adaptation of LAB to nutrient-rich and extreme environments.

Beside their major nutritional characteristics, sugar-rich environments can inhibit or prevent bacterial growth and cell division due to the presence of chaotropic solutes (e.g., phenols, ethyl acetate, ethanol, glycerol, fructose) and hydrophobic stressors (such as hexane, ethyl octanoate, or octanol acetate) (Lievens et al. 2015). Fructophilic lactic acid bacteria (FLAB) described recently by Endo and coworkers were found to possess the ability to invade niches rich in high concentrations of sugar, especially fructose (Endo and Okada 2008). They can be found in such environments as flowers, nectar, fruits, and in regional foods like tempoyak (made mainly from fermented durian) or taberna (alcoholic beverage) (Endo et al. 2009). Fructophilic LAB have also been discovered in the digestive tracts of pollinators such as bees, bumblebees, or in general in insects consuming significant amounts of fructose, e.g., tropical fruit flies or ants from the genus *Camponotus.* Ants willingly feed on honeydew, which is a mixture of fructose-rich juices of plants damaged by aphids and the liquid excrement of these insects. *Fructobacillus fructosus* isolated from a flower in Japan was described as FLAB for the first time (Endo and Okada 2008). Subsequent papers described instances of *Fructobacillus* from South Africa, Mexico, or the USA. To the best of the authors’ knowledge, there have been no reports on *Fructobacillus* from Eastern Europe (Antunes et al. 2002; Endo et al. 2010, 2012; Endo 2012).

The group of FLAB includes five species from the genus *Fructobacillus* and two species from the genus *Lactobacillus.* The genus *Fructobacillus* prefers D-fructose to D-glucose as a main source of growth, due to the absence of the *adhE* gene encoding a bifunctional alcohol/acetaldehyde dehydrogenase. For glucose metabolism, *Fructobacillus* species require fructose, oxygen, or pyruvate as an external electron acceptor due to the shortage of NAD+ (Endo et al. 2014). Under anaerobic conditions where glucose is the only carbon source, the bacteria show no or very poor growth. This description applies to “obligately” fructophilic lactic acid bacteria, distinguished in the group of FLAB according to the two types of sugar metabolism. *Fructobacillus fructosus*, *F. ficulneus*, *F. pseudoficulneus*, *F. durionis*, *F. tropaeoli*, and *Lb. kunkeei* are classified as “obligately” fructophilic bacteria*. Lb. florum* represents the group of “facultatively” fructophilic lactic acid bacteria*.* “Facultatively” fructophilic bacteria can grow on glucose without an external electron acceptor and produce ethanol from glucose; however, the growth of FLAB on fructose is faster (Endo et al. 2012). Fructophilic lactic acid bacteria can also produce polyols such as glycerol, erythritol, or mannitol (Endo and Okada 2008; Endo and Dicks 2014; Tyler et al. 2016).

As core members of the microbiome of honeybees and other pollinators, FLAB are currently investigated for their influence as potential probiotics (Endo and Salminen 2013; Vojvodic et al. 2013). Some FLAB have antibacterial activity against *Paenibacillus larvae* and *Melissococcus plutonius* causing foulbrood diseases (Forsgren et al. 2010; Rokop et al. 2015). These bacteria are capable of utilizing more complex carbohydrates than fructose and glucose, such as lignin. Degradation of lignin, which is a component of pollen, by these bacteria helps to utilize this vital bee food (Alberoni et al. 2016). Therefore, it is believed that *Fructobacillus* bacteria can be syntrophic through the distribution/decomposition of more complex compounds and enhancement of their availability to other microbiome bacteria (Rokop et al. 2015).

The first aim of this study was to isolate and identify fructophilic lactic acid bacteria from honeydew. The second aim of our work was to characterize some biological properties of a newly isolated fructophilic *Lb. plantarum* FPL strain.

## Materials and methods

### Isolation of fructophilic lactic acid bacteria

Honeydew produced by *Coccus hesperidum* L. was collected in gardens in Lublin, Poland, Eastern Europe in August 2015. Honeydew samples were placed in sterilized Eppendorf tubes with saline. The samples were incubated for 1 h with shaking on a heating ThermoMixer HLC (DITABIS AG, Pforzheim, Germany) at 30 °C and 1000 rpm. This solution was transferred to a FYP (fructose yeast peptone) medium (Endo et al. 2015) and MRS with fructose (2% (*w*/*v*)). The inoculated medium was incubated at 30 °C for 24 h in aerobic conditions; then, it was moved onto Petri plates on MRS with fructose and FYP. When colonies were visible, they were selected in terms of their morphological properties (shape, size, color). To obtain pure cultures, the colonies were isolated by streaking on agar plates.

### Identification of isolates using MALDI-TOF

Forty-nine isolates of bacteria were identified using the MALDI-TOF Biotyper (Bruker Daltonics, Bremen, Germany). *Lb. plantarum* NRRL B-4496 (ARS Culture Collection, Peoria, IL, USA) was used as a reference strain. After a 24-h incubation, a single colony was transferred to an Eppendorf tube with 150 μl of sterile deionized water. The samples were homogenized by repeated pipetting and vortexing. Four hundred fifty microliters of pure ethanol were added to the Eppendorf, and the content was mixed by vortexing for at least 1 min. After centrifugation for 2 min at 13000 rpm, the supernatant was removed; this step was repeated twice. A 70% solution of formic acid was added in an amount of 40 μl and vortexed, and the same volume of 99% acetonitrile was added as well. After vortexing for 1 min, the samples were centrifuged (2 min, 13,000 rpm). One microliter of the supernatant was applied onto a metal plate in triplicate. After drying at room temperature, the spots were covered with 1 μl of matrix (concentration of 10 mg of HCCA- α-Cyano-4-hydroxycinnamic acid/ml) and left to dry. The plate was introduced to an UltrafleXtreme MALDI TOF mass spectrometer (Bruker, Germany) with a 1000 Hz neodymium-doped yttrium aluminum garnet nitrogen laser (Nd-YAG). The samples were analyzed automatically using a MALDI Bio-typer 3.0 software package (Bruker, Germany). The probability of identification was expressed by a score in a scale from 0 to 3.0. A result above 2.0 denoted secure genus identification and probable species identification. Nine isolates were selected based on the high probability of identification for further experiments in this article.

### 16S rRNA gene sequencing and species-specific PCR

DNA extraction of nine strains was performed using Genomic Mini AX Bacteria Spin (A&A Biotechnology, Gdynia, Poland) according to the attached protocol. For amplification of the 16S rRNA gene, universal primers (27f) 5`-AGAGTTTGATCCTGGCTCAG-3`, and (1495r) 5`-CTACGGCTACCTTGTTACGA-3` were used (Genomed S.A., Warszawa, Poland). The PCR reaction was performed in a total volume of 20 μl using a PCR Master Mix(2×) (Thermo Fisher Scientific, Bermen, Germany) in a Labcycler (SensoQuest Göttingen, Germany). The amplification reaction was characterized by the following steps in 30 repeat cycles: denaturation 95 °C for 1 min, annealing 48 °C for 30 s, elongation 72 °C for 2 min, final extension 72 °C for 10 min, and cooling the samples to 4 °C. The amplification products were separated on 1.5% agarose gel (Eurx, Gdańsk, Poland). The nucleotide sequences were determined by the BigDye Terminator v3.1 Cycle Sequencing Kit (Applied Biosystems, USA), and the capillary sequencing system, 3730xl DNA Analyzer (Applied Biosystems, USA). Sequences were assembled by a DNA Baser Assembler, subsequently aligned with BLAST, and compared in the NCBI GenBank to find the closest relatives. A neighbor-joining tree was made using MEGA 4 for the phylogenetic analysis based on 16S rRNA sequences. Only one representative sequence was used to create the diagram, because there was no differentiation after alignment. Sequences of the 16S rRNA gene used to construct the phylogenetic tree were approximately 1450 base pairs.

Additionally, multiplex PCR, which detected the *rec*A gene phylogenic marker, was used; this revealed distinction between *Lb. plantarum*, *Lb. pentosus*, and *Lb. paraplantarum* (Torriani et al. 2001). Multiplex was performed with four primers paraF (59-GTC ACA GGC ATT ACG AAA AC-39), pentF (59-CAG TGG CGC GGT TGA TAT C-39), planF (59-CCG TTT ATG CGG AAC ACC TA-39), and pREV (59-TCG GGA TTA CCA AAC ATC AC-39). The composition of the reaction was 13 μl PCR Master Mix(2×) (Thermo Fisher Scientific, Bermen, Germany), 0.75 μl for each primer, and 10.5 μl nuclease-free water (Thermo Fisher Scientific, Bermen, Germany); the reaction conditions were described previously (Torriani et al. 2001). The amplification products were separated on 1.5% agarose gel (Eurx, Gdańsk, Poland) with 1 kb Ladder Perfect Plus (Eurx, Gdańsk, Poland). In order to clarify fructophilic properties, a PCR reaction of the adhE gene was performed; the reaction conditions were described previously (Maeno et al. 2016).

### Biochemical characterization

Carbohydrate fermentation was determined with a Hi-Carbo Kit (HiMedia, Mumbai, India). An inoculum with turbidity 0.5 OD nm at 600 nm was added onto wells containing 35 sugars and incubated at 37 °C for 24 and 48 h. For carbohydrate utilization, a reference strain *Lactobacillus plantarum* NRRL B-4496 was additionally used. Gas production from glucose was read with the Durham test, and catalase activity was determined by reaction with 3% (*v*/*v*) H_2_O_2_. API ZYM (bioMérieux SA, Marcy l′Etoile, France) was used for determination of enzyme production patterns. An inoculum with turbidity 0.8 OD nm at 600 nm was added onto 20 plates and incubated for 4 h at 37 °C.

### Biological activity

#### Fructophilic properties of the isolates

Fructophilic properties were determined using a Bioscreen C system (Labsystem, Helsinki, Finland). After a 24-h incubation, bacterial cultures were centrifuged and removed from the medium. The bacterial cells were suspended in physiological saline, and the same optical density of 0.5 was set at 600 nm. The analyzed bacteria were grown in FYP with 10 g (L^−1^) D-fructose, GYP with 10 g (L^−1^) D-glucose, and GYP-P with 5 g (L^−1^) D-glucose and 5 g (L^−1^) pyruvate as an external electron acceptor, and MRS with 300 g (L^−1^) D-fructose, with 300 g (L^−1^) D-glucose, with 400 g (L^−1^) D-glucose, and with 500 g (L^−1^) D-glucose. Three hundred fifty microliters of the media were transferred onto honeycomb 100-well plates in triplicate, and the wells were inoculated with 50 μl of the bacterial suspension. The experiment was performed in aerobic and anaerobic conditions by measuring the OD_600nm_ every 2 h for 48 h. Anaerobic conditions were obtained by cutting off access to oxygen with a few drops of paraffin. Based on the growth characteristics, nine strains were chosen for further examination. Growth curve parameters (max specific growth rate, lag time, doubling time, etc.) were determined using the PYTHON script according to Hoeflinger et al. (2017). High sugar tolerance was tested in FYP and MRS broth enriched with 20, 30, 40, and 45% (*w*/*v*) fructose and glucose or containing 5% of NaCl (w/*v*) by observing a significant amount of biomass in the probe. Production of lactic acid was checked by incubation on FYP-agar and MRS-agar containing 10 g (L^−1^) CaCO_3_ and confirmed with HPLC with a UV-Vis detector (Gilson Medical Electronics, Villiers-le-Bel, France). Production of sugars was determined after 3-day culture in rotary shaker with aeration (150 rpm) (Minitron Incubator Shaker Infors AG, Bottmingen, Switzerland). The amount of glucose and fructose consumed after 3 days of incubation was determined using an IR detector. A reference strain *Lactobacillus plantarum* NRRL B-4496 was used as a control in all tests.

### Antibiotic susceptibility test

After 24 h incubation in 30 °C, the cells were centrifuged and removed from the culture medium with saline. The inoculum suspension in saline with McFarland density of 0.5 was carefully spread on Petri plates with 4-mm thick MRS agar. When the suspension was absorbed by the agar, rings with antibiotics were distributed in triplicate. Erythromycin E15, kanamycin K30, bacitracin B10, streptomycin S10, amoxycillin AML25, tetracycline TE30, trimethoprim WE, penicillin P10, pirlimycin PIR2, chloramphenicol C30, and nalidixic acid NA30 were purchased from Oxoid (Hampshire, England).

### Statistical analysis

The values from all measurements are mean ± standard deviation. The data were analyzed using the Excel statistical package. Statistical significances were determined by Student’s *t* test and set at *P* =w0.01.

### GeneBank accession number

GeneBank accession number for *Lb. plantarum* FPL 16S rRNA gene: KY883188. Due to the sequence identity obtained for the 16S rRNA gene, only one representative of this group was included.

## Results and discussion

### Species identification by MALDI-TOF

The identification of 49 strains was performed by MALDI-TOF, and the spectra obtained were aligned with the Brucker database. The spectra of the isolated strains had a high probability of identification over two points, (experiments were carried out in triplicate). Analysis of 46 strains spectra indicated *Lb. plantarum,* three other spectra corresponded to *Staphylococcus haemolyticus*, *S. aureus*, and *Enterococcus mundtii*. According to the Brucker database, almost all isolates indicated *Lb. plantarum*; only three isolates showed other species, but they can be considered as contamination. The results showed the dominance of *Lb. plantarum* in the honeydew environment. Subsequently, spectra of the new FPL strains were aligned with the reference strain, and the shift of some peaks indicates modification of proteins. The MALDI Biotyper analysis of the spectra shows that the surface of certain proteins was modified, which may explain the adaptation to the fructose-rich environment. The results of the MALDI-TOF analysis revealed nine isolates preliminary identified as *Lb. plantarum,* with the highest score of probability of identification.

### Species identification through 16S rRNA gene sequencing and multiplex PCR

**Fig. 1:**
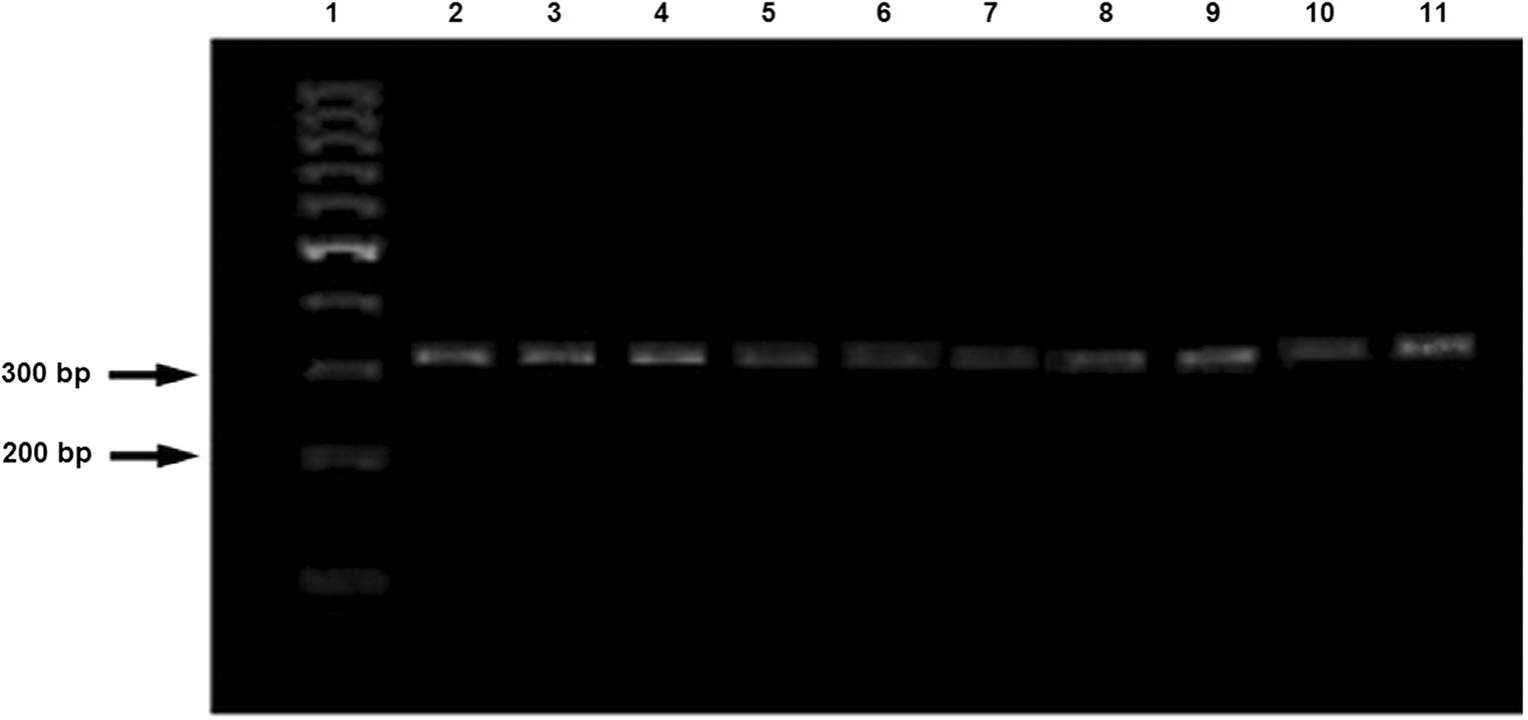
PCR amplification products obtained from the multiplex assay. Lane 1 contains a 1 kb Ladder Perfect Plus (Eurx, Gdańsk, Poland). Lane 2 contains the amplification product from *Lb. plantarum* FPL with a length of 295.53 bp; lane 3 shows amplification products from *Lb. plantarum* FPL1 with a length of 295.56 bp; line 4 *Lb. plantarum* FPL2 295.6 bp; line 5 *Lb. plantarum* FPL3 298 bp; line 6 *Lb. plantarum* FPL4 298.5; line 7 *Lb. plantarum* FPL5 294 bp; line 8 *Lb. plantarum* FPL6 296 bp; line 9 *Lb. plantarum* FPL7 299 bp; line 10 *Lb. plantarum* FPL8 308 bp; line 11 *Lb. plantarum* FPL9 310 bp. The length/number of base pairs was determined using Quantity One Software (Bio-Rad, Illinois, USA)

**Fig. 2:**
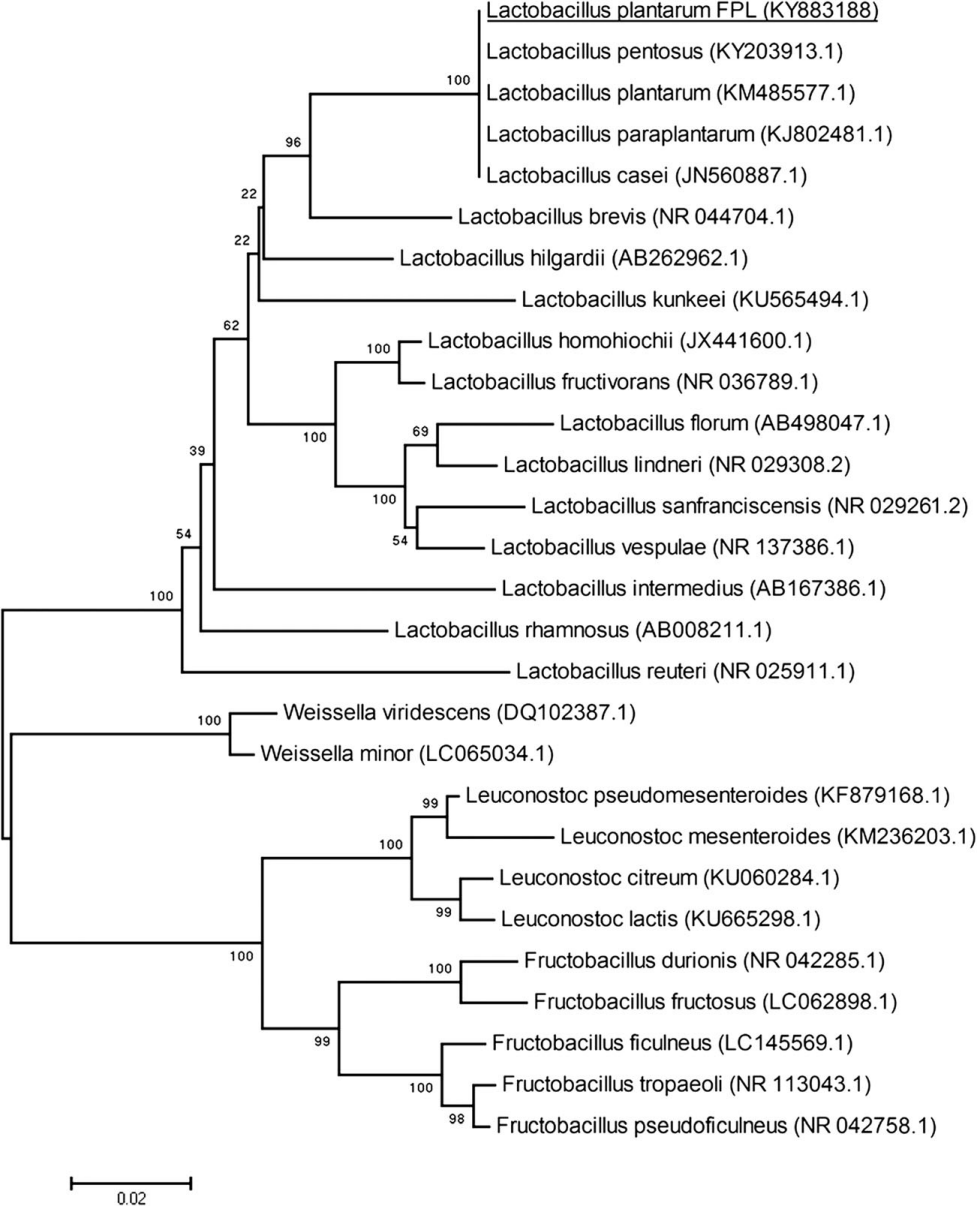
Phylogenetic tree based on the sequence of 16S rRNA showing the relative positions of *Lb. plantarum* FPL

The identification of nine isolates of *Lb. plantarum* from honeydew was performed by analyzing sequences of the 16S rRNA gene. The DNA sequences obtained were aligned by BLAST with the nucleotide gene bank; it was revealed that all strains are > 99% similar to *Lb. plantarum*, *Lb. paraplantarum*, and *Lb. pentosus*. In order to confirm the species belonging of the isolates, a multiplex PCR was performed. Reaction products of multiplex PCR for *rec*A gene with length of about 310 bp is specific to the *Lb. plantarum* species (Fig. 1). The phylogenetic tree constructed with the neighbor-joining method shows strains that are the closest to *Lb. plantarum* FPL as well as the location of *Fructobacillus* species. *Lactobacillus kunkeei* is the nearest phylogenetic neighbor from the group of FLAB (Fig. 2)*.* In the first reports on FLAB, growth on various media was described; the 16S rRNA gene was identified, and a few biochemical and fructophilic properties were characterized (Endo and Okada 2008). In this article, the studies proposed by Endo were conducted. We also used MALDI-TOF and multiplex PCR which made it possible to identify strains from the honeydew. All these methods facilitated quick and efficient selection of strains for further research.

### Biochemical properties

The *Lb. plantarum* FPL strains utilize carbohydrates indicated in Table 1. The different strains are able to use also xylose, galactose, raffinose, glycerol, and adonitol, which may indicate the dissimilarity of individual isolates. The reference strain *Lb. plantarum* NRRL B-4496 showed no differences in utilization of carbohydrates, except inulin, raffinose, and mannitol, which were used only by the fructophilic *Lb. plantarum* FPL.

**Table 1:** Carbon utilization profile of Lb. plantarum FPL and Lb. plantarum NRRL B-4496 determined with the Hi-Carbo Kit (HiMedia, Mumbai, India), + positive (+++clearly visible change, ++ visible, + poorly visible); − negative

Carbon source	Lb. plantarum FPL	Lb. plantarum FPL1	Lb. plantarum FPL2	Lb. plantarum FPL3	Lb. plantarum FPL4	Lb. plantarum FPL5	Lb. plantarum FPL6	Lb. plantarum FPL7	Lb. plantarum FPL8	Lb. plantarum NRRL
Lactose	+	++	++	++	++	++	+	++	++	++
Xylose	+	–	–	–	–	–	–	–	–	+
Maltose	+++	++	++	+++	+++	+++	+++	++	+++	++
Fructose	+++	+++	+++	+++	+++	+++	+++	+++	+++	+++
Dextrose	+++	+++	++	+++	+++	+++	+++	+++	+++	+++
Galactose	–	+	+	+	–	+	+	+	+	+
Raffinose	+	–	+	–	–	–	–	–	–	–
Trehalose	++	+++	++	+	+	++	++	++	++	+
Sucrose	+	++	+++	++	++	++	+++	++	++	++
Mannose	+++	+++	+++	+++	+++	+++	+++	++	+++	+++
Inulin	+++	+++	+++	++	+++	+++	+++	+++	+++	–
Salicin	++	+++	++	++	++	+++	+++	++	++	++
Sorbitol	+	+	+	+	+	+	+	+	+	+
Mannitol	+	++	+	+	++	+	+	+	+	–
Cellobiose	+++	+++	+++	+++	+++	+++	+++	+++	++	+++
Melezitose	++	++	+++	++	++	++	++	++	++	++
α-methyl-D-mannoside	+	+	+	++	++	+	++	+	+	++
Esculin	+++	+++	+++	+++	+++	+++	+++	+++	+++	+++

The results of API ZYM (Biomerieux) revealed production of esterase (C4), esterase lipase (C8), lipase (C14), leucinearylamidase, valinearylamidase, cystine arylamidase, acid phosphatase, naphthol AS-BI-phosphohydrolase, β-galactosidase, α-glucosidase, β-glucosidase, and N-acetyl-β-glucosaminidase. The strains had no activity of alkaline phosphatase, trypsin, α-chymotrypsin, α-galactosidase, β-glucuronidase, α-mannosidase, and α-fucosidase. The study conducted by Siezen et al. (2010) tested carbohydrate utilization by 185 strains of *Lb. plantarum*; all strains degraded trehalose, sucrose, melezitose (except one), and sorbitol similarly to the *Lb. plantarum* FPL strains. There were differences in the case of mannitol and inulin; this study has shown that only the *Lb. plantarum* FPL strains utilize these carbohydrates, which often occur in the plant environment. However, utilization of inulin by the *Lb. plantarum* species is not unique, as other strains have been reported to degrade grass fructan and inulin (Müller and Steller 1995; Siezen et al. 2010; Valan Arasu et al. 2015). In contrast, the possibility of different carbohydrate metabolism by the *Lb. plantarum* FLP strains is significantly higher than that of the FLAB group, as the latter bacteria do not degrade salicin, sorbitol, cellobiose, and melezitose (Endo et al. 2010, 2012; Lievens et al. 2015). All species in the FLAB group can metabolize mannitol and fructose, as same as fructophilic properties of the *Lb. plantarum* FPL strains (Endo and Okada 2008). The strains do not exhibit acid phosphatase, trypsin, and chymotrypsin activity, which is present in most *Fructobacillus* species. *Lb. plantarum* FPL has β- galactosidase, α- glucosidase, and β- glucosidase activity, unlike the genus *Fructobacillus*, which may cause syntrophic interactions between these bacteria through metabolic by-products.

### Antibiotic susceptibility test

The antibiotic sensitivity slightly differs between the individual strains, as shown in Table 2. The strains are sensitive to all antibiotics used in this study except nalidixic acid. The antibiotic-sensitivity test has shown that the *Lb. plantarum* strains are safe which is the first step to determining their probiotic potential. The antibiotic-susceptibility test was carried out earlier on FLAB that are inextricably linked to insects and have probiotic potential for insects. Furthermore, FLAB can produce and utilize substances supporting the growth of the core gut microbiome of honey bees (Rokop et al. 2015).

**Table 2:** Antibiotic sensitivity of nine isolated *Lb. plantarum* FPL

Antibiotic	Concentration of antibiotic	Average zone of inhibition A (mm) and Standard deviation SD(mm)																			
		FPL		FPL1		FPL2		FPL3		FPL4		FPL5		FPL6		FPL7		FPL8		Total	
		A	SD	A	SD	A	SD	A	SD	A	SD	A	SD	A	SD	A	SD	A	SD	A	SD
Eryhtomycin E15	15 μg	30.37	0.26	28.83	0.24	30.67	0.94	22.67	0.47	19.67	0.47	26.33	0.47	29.67	0.62	29.03	0.73	26.60	1.18	27.09	0.29
Kanamycin K30	30 μg	10.17	0.24	9.37	0.26	8.00	0.71	8.33	0.47	9.87	0.19	8.30	1.28	7.00	0.00	8.10	0.54	9.50	0.71	8.74	0.36
Bacitracin B10	10 units	21.87	0.66	22.83	1.43	11.67	0.47	16.00	1.41	17.50	0.41	13.17	3.06	20.67	1.25	19.83	0.62	19.20	1.57	18.08	0.78
Streptomycin S10	10 μg	12.83	0.62	14.93	0.33	8.50	0.41	10.33	0.47	10.50	0.71	12.17	0.85	9.90	1.58	11.17	1.43	9.50	1.87	11.09	0.53
Amoxycillin AML25	25 μg	37.60	0.22	36.20	0.59	27.50	1.22	30.33	3.77	31.67	1.25	29.33	0.47	30.83	0.85	34.00	0.82	31.67	2.49	32.13	1.07
Tetracycline TE30	30 μg	22.10	0.54	22.60	1.13	17.00	2.45	15.67	0.47	20.53	0.41	24.83	0.62	23.17	1.93	18.83	1.65	21.00	1.63	20.64	0.70
Trimethoprim WE	5 μg	25.13	0.81	25.00	0.00	24.50	1.08	25.00	0.00	22.97	0.82	21.33	1.43	19.00	0.71	20.27	0.21	20.50	0.41	22.63	0.46
Penicillin P10	10 units	24.17	0.24	26.63	0.45	24.00	0.82	20.00	0.00	22.83	0.62	20.67	3.30	19.00	0.82	25.47	0.34	19.00	2.16	22.42	1.01
Pirlimycin PIR2	2 μg	8.33	0.47	9.83	1.18	21.67	0.47	11.83	0.62	8.17	0.62	8.30	0.99	10.33	0.47	9.17	0.24	10.00	0.82	10.85	0.28
Chloramphenicol C30	30 μg	31.67	0.47	21.83	1.43	23.83	0.85	23.00	0.71	31.67	0.94	29.23	1.58	22.83	1.65	24.67	2.62	31.00	1.41	26.64	0.61
Nalidixic acid NA30	30 μg	0.00	0.00	0.00	0.00	0.00	0.00	0.00	0.00	0.00	0.00	0.00	0.00	0.00	0.00	0.00	0.00	0.00	0.00	0.00	0.00

### Fructophilic properties

**Fig. 3:**
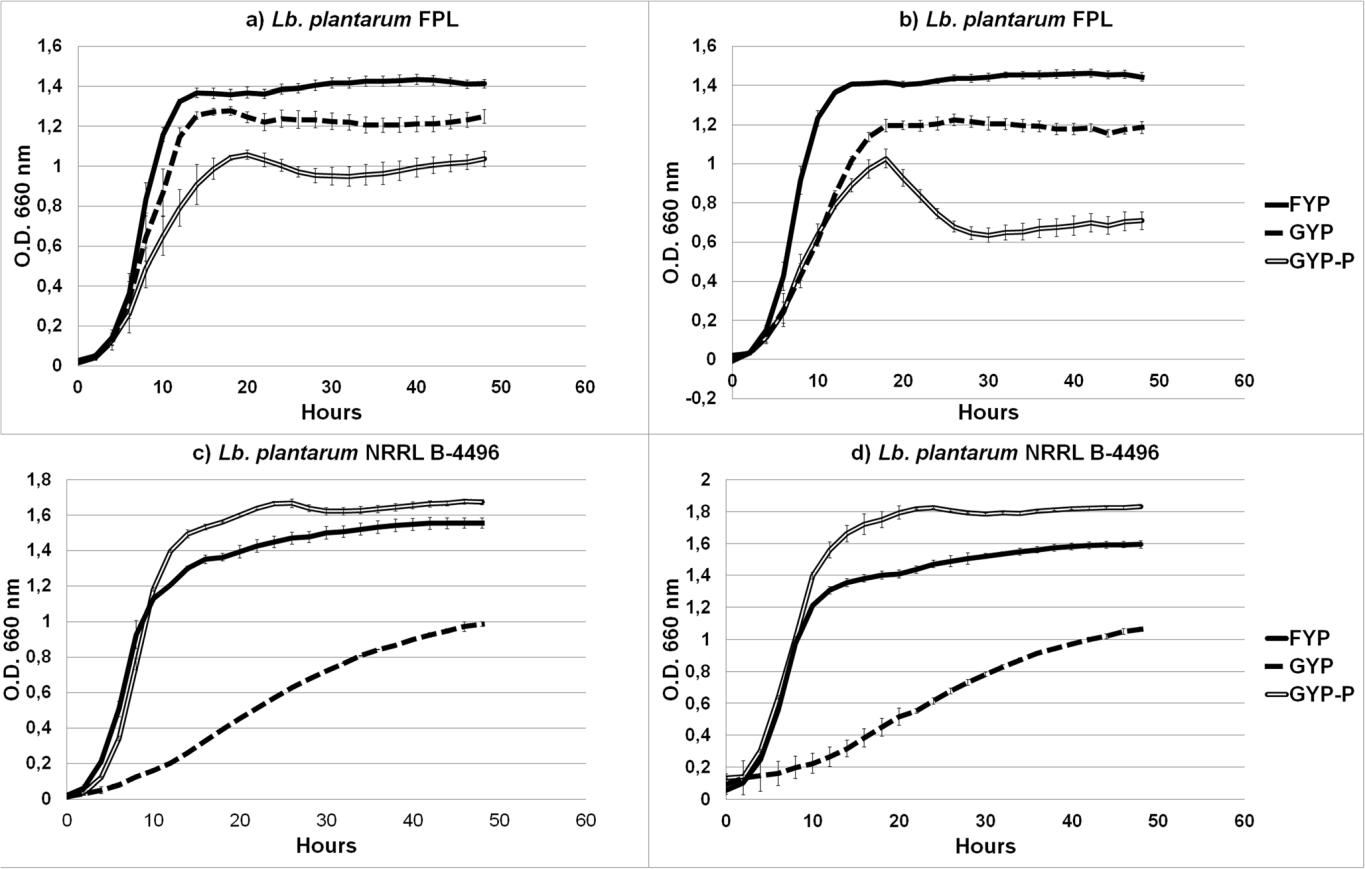
Growth curves of Lb. plantarum FPL in **a** aerobic conditions, **b** anaerobic conditions and of Lb, plantarum NRRL B-4496 in **c** aerobic conditions, and **d** anaerobic conditions on various carbon sources (FYP, GYP, GYP-P)

All tested carbon sources caused a considerable growth with the used bacteria (i.e., reached a final OD 660 > 0.9). Strains isolated in this study, showed very similar growth curves; therefore, only one representative strain is shown in Fig. 3. Growth parameters are shown in Table 3. However, distinctive growth profiles were obtained within one species, where sugar preferences are clearly visible depending on the strain. The comparison of the growth curves of *Lb. plantarum* FPL and *Lb. plantarum* NRRL-4496 shows different preferences for sugars as a growth substrate. The max specific growth rate of *Lb. plantarum* FPL on fructose was highest in the entire study. The origin of the *Lb. plantarum* FPL strains had to determine their tendency towards fructose, which is a significant component of honeydew. Strains of *Lb. plantarum* FPL grew fast on FYP, both under anaerobic and aerobic conditions, which allowed us to state its fructophilicity, which is a niche-specific adaptation. Reference strain NRRL- 4496 does not exhibit such affinity for fructose.

**Table 3:** Growth parameters of *Lactobacillus plantarum* FPL and *Lactobacillus plantarum* NRRL B-4496

Medium	Strain	Growth conditions	Lag time (hours)	Max specific growth rate (hours^−1^)	Doubling Time (hours)	Max OD	Max OD (median filtered data)	Min. OD	Min. OD (median filtered data)	Delta OD (median filtered data)	*R* ^2^	RMSE (root-mean-square error)
GYP	NRRL	Aerobic	5.2473	0.2059	3.3657	1.6777	1.6760	0.0127	0.0127	1.6633	0.9970	0.0299
GYP	FPL	Aerobic	3.6182	0.0962	7.2084	1.0590	1.0508	0.0177	0.0177	1.0332	0.9990	0.0125
GYP	NRRL	Anaerobic	3.9524	0.1951	3.5537	1.8303	1.8303	0.1063	0.1327	1.6977	0.9981	0.0235
GYP	FPL	Anaerobic	4.2491	0.1010	6.8595	1.0267	1.0002	0.0153	0.0153	0.9848	0.9990	0.0122
FYP	NRRL	Aerobic	0.5822	0.1344	5.1568	1.5563	1.5550	0.0227	0.0227	1.5323	0.9870	0.0531
FYP	FPL	Aerobic	5.2862	0.2143	3.2347	1.4330	1.4313	0.0273	0.0273	1.4040	0.9973	0.0228
FYP	NRRL	Anaerobic	1.9264	0.1497	4.6312	1.5933	1.5933	0.0553	0.0553	1.5380	0.9836	0.0593
FYP	FPL	Anaerobic	4.9179	0.2244	3.0892	1.4630	1.4617	− 0.0063	− 0.0063	1.4680	0.9983	0.0181
GYP-P	NRRL	Aerobic	2.5441	0.0305	22.7309	0.9880	0.9880	0.0160	0.0160	0.9720	0.9995	0.0075
GYP-P	FPL	Aerobic	5.0423	0.1594	4.3497	1.2773	1.2750	0.0157	0.0157	1.2593	0.9986	0.0184
GYP-P	NRRL	Anaerobic	4.7016	0.0302	22.9741	1.0637	1.0637	0.0937	0.0937	0.9700	0.9991	0.0099
GYP-P	FPL	Anaerobic	5.2819	0.1141	6.0773	1.2243	1.2207	0.0220	0.0220	1.1987	0.9991	0.0134

Another significant difference between the strains is growth on the medium supplemented with pyruvate. Pyruvate as well as oxygen, citrate, and fructose can be used by LAB as external electron acceptors (Zaunmüller et al. 2006), so Endo et al. used it in their experiments to show the characteristic properties of fructophilic lactic acid bacteria (Endo et al. 2009, 2015). Our observations indicate that pyruvate, also stimulates the growth of *Lb. plantarum* FPL on glucose, especially with aerobic conditions. It is known that *Lb. plantarum* grows better in aerobic conditions and what is more, it is able to use oxygen as a substrate (Zotta et al. 2012). Moreover, in the presence of oxygen, the expression of genes responsible for the consumption of sugars increases (Guidone et al. 2013; Zotta et al. 2013). Successively both in aerobic and anaerobic conditions, the growth of the reference NRRL-4496 strain, on the pyruvate medium, is slower compared to our FPL strain. This can be explained by the fact that NRRL-4496 strain does not exhibit fructophilic properties. Furthermore, the latest articles report that *Lb. plantarum* species can use pyruvate in various metabolic pathways (Zotta et al. 2017).

Although strain NRRL B-4496 grows on fructose, it is not able to grow on a medium with a high concentration of both glucose and fructose. The reference strain did not develop mechanisms that would allow survival in a sugar-rich environment. The growth curves of FLAB already described (Endo et al. 2009) are lower in comparison with *Lb. plantarum* FPL. The experiments conducted in this article show that MALDI-TOF and Bioscreen C facilitate rapid screening of fructophilic lactic acid bacteria.

**Fig. 4:**
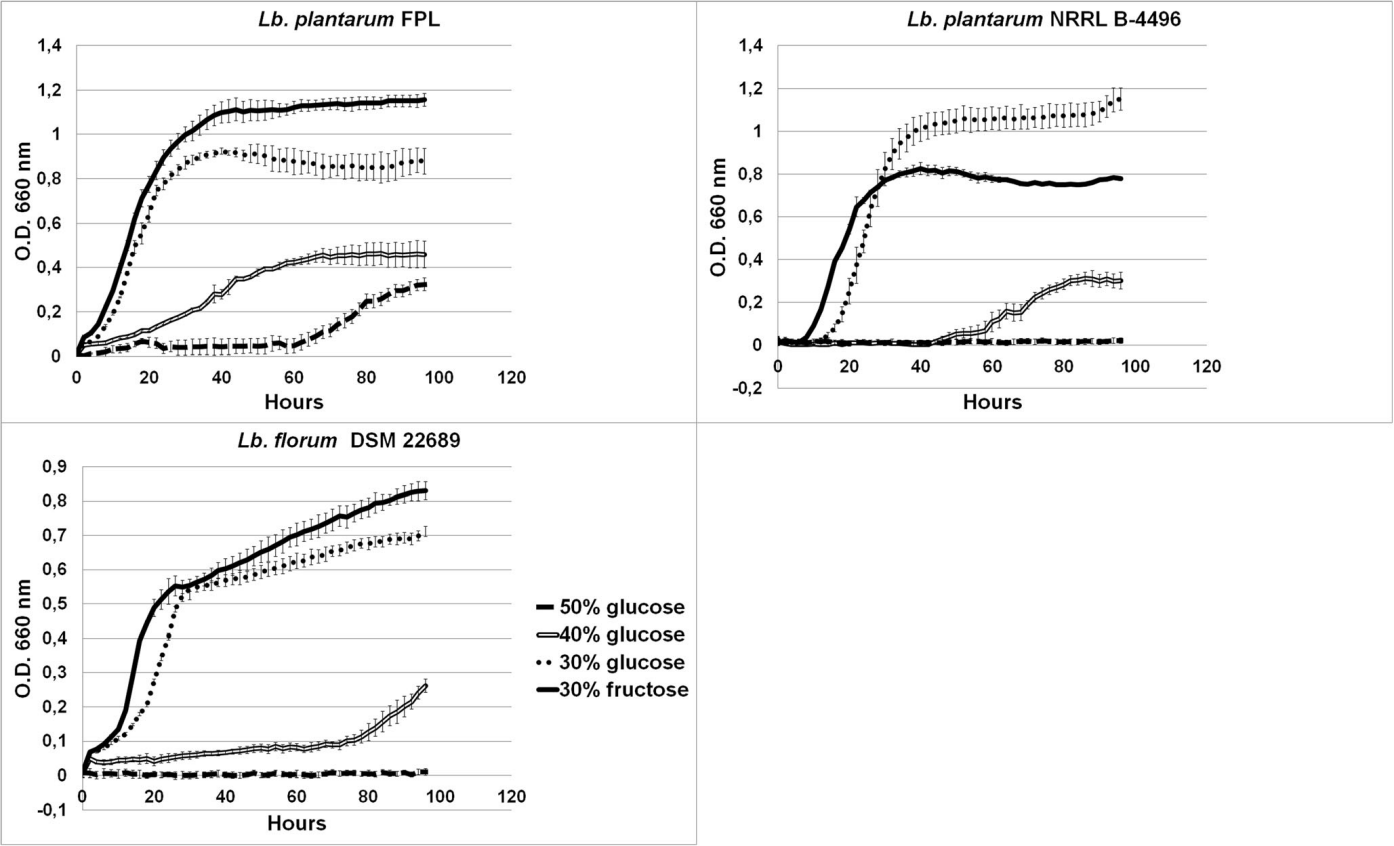
Growth curves of *Lb. plantarum* FPL, *Lb. plantarum* NRRL-4496, *Lb. florum* DSM on various medium with high sugar concentrations 30, 40, and 50% of glucose or fructose in aerobic conditions

In addition, the newly described strain can grow on a medium with 50% (*w*/*v*) fructose; other FLAB, except *F. tropaeoli*, tolerate 40% (w/v) fructose content. The growth curves in Fig. 4 show the adaptation to growth in high sugar concentrations. In the case of the *Lb. plantarum* FPL strain, the fructophilicity are again visible. The strain grows best on a medium with a concentration of 30% fructose, then 30% glucose. A slow growth rate can be seen on the medium with 40% glucose, and even delayed on medium with 50% glucose. This osmotolerance is high, since generally bacteria and yeast tolerate up to 50% (*w*/*v*) of sugar (Álvarez-Pérez et al. 2012).

The tolerance to high concentrations of sugar in the MRS and FYP media containing 20, 30, 40, 45, and 50% (*w*/*v*) of glucose or fructose was evidenced by the presence of significant amounts of biomass at the bottom of the tube. The bacterial growth was visible as a biomass after 24 h of incubation on broth with the 20 and 30% concentrations of glucose or fructose. After 48 h, the growth was visible in the medium with the 40, 45, and 50% sugar concentration; in addition to the biomass at the bottom, there was evident turbidity. The difference between the growth on fructose and glucose was not significant. A transparent zone, which indicated production of acids, appeared around the colonies on MRS and FYP agar with CaCO3. In the supernatant, 3.4% of lactic acid was detected by HPLC. The tested bacteria produced gas from glucose and were catalase-negative. HPLC detected that the cultured *Lb. plantarum* FPL strain produced glycerol from fructose; no polyols were detected. In addition, HPLC confirmed that the strain utilized both carbon sources but first fructose.

**Fig. 5:**
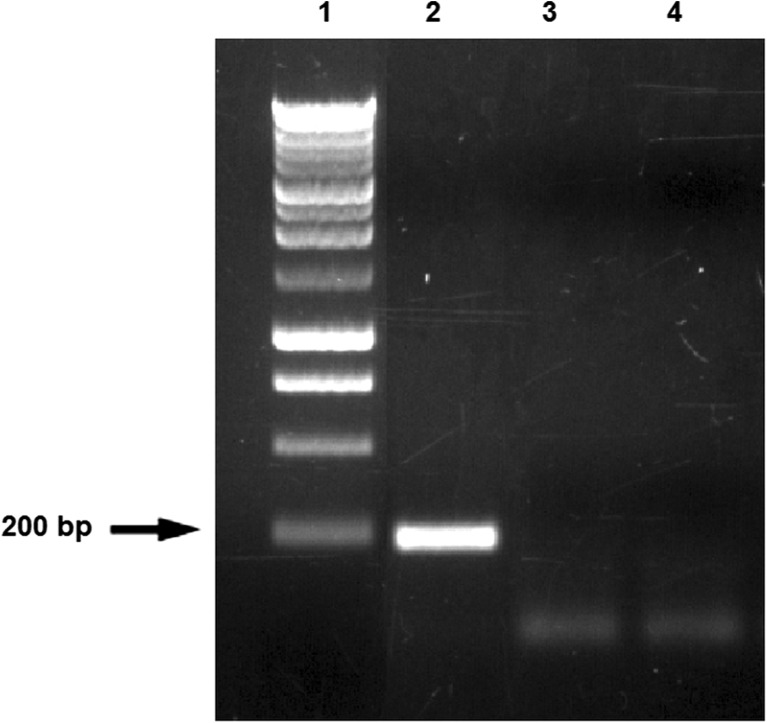
PCR amplification products obtained with *adhE* primers. Lane 1 contains a 1 kb Ladder Perfect Plus (Eurx, Gdańsk, Poland). Lane 2 contains the amplification product from *Lb. florum* DSM 22689; lane 3 shows amplification products from *Lb. plantarum* FPL; line 4 *Lb. plantarum* NRRL B-4496

Many FLAB, as well as *Lb. plantarum* FPL, were isolated with the use of FYP containing 30% fructose. It was also noted that more isolates were cultured on FYP than on MRS with fructose. This confirms that FYP is a specialized media in which a high concentration of fructose selects FLAB and simultaneously inhibits growth of other bacteria (Le Marrec et al. 2007; Endo et al. 2009). The absence of fructose in commercial media explains why fructophilic bacteria were not identified earlier (Endo 2012). *Lb. plantarum* strains are widespread in various environments, probably thanks to one of the largest genomes among lactic acid bacteria. Generally Lactobacilli have a relatively large number of transport and regulatory genes as well as sugar transport and utilization genes (Álvarez-Pérez et al. 2012). In the genome of *Lb. plantarum* WCFS1, 30 sugar transport systems have been found, which explains why this species can grow on a variety of carbon sources. Among the genes of *Lb. plantarum* that are most expressed besides housekeeping genes, there are genes of the Embden–Meyerhoff–Parnas (EMP) pathway and many genes encoding enzymes involved in pentose and hexose utilization. The sequencing of the whole genome showed that potentially highly expressed (PHX) genes included numerous of phosphotransferase systems (PTSs), especially fructose and mannose PTS systems (Kleerebezem et al. 2003). This flexibility may explain the appearance of the fructophilic properties of the *Lb. plantarum* FPL strain. Moreover, in this article, we have described strains that prefer fructose as a source of growth, with resistance to high sugar concentrations. If the extended *Lb. plantarum* genome, which has allowed adaptation to the fructose rich environment, is the cause of the fructophilic properties of the new FPL strains, this stands in opposition to the origin of the fructophilic characteristics of FLAB. Adaptation to fructose in the FLAB group is due to the lack of the *adhE* gene; in the case of *Lb. plantarum* FPL, the mechanism of the fructophilic behavior has a different basis (Fig. 5). Certainly, in part, this is explained by the huge number of genes responsible for metabolism and transport of sugars in the genome, but the question remains why this strain prefers fructose instead of glucose as opposed to *Lb. plantarum* NRRL. The presence of *Lb. plantarum* in honeydew must have an impact on the ecosystem of aphids, bees, or ants. It is also very possible that *Lb. plantarum* inhabit the digestive tracts of aphids and honeydew-consuming insects. Yeasts living in nectar increase the number of visits of pollinators (Herrera et al. 2013), while the presence of certain bacteria in the nectar (*Erwinia tasmaniensis*, *Lactobacillus kunkeei*, *Asai astilbes*) repel insects from flowers by changing the chemical composition of the nectar (Good et al. 2014). More samples of honeydew from Poland should be investigated to confirm the colonization of this habitat by *Lb. plantarum* and its effect on insects.

### Data availability statement

All data generated or analyzed during this study are included in this published article

## Conclusion

The main goal of this work was to isolate fructophilic lactic acid bacteria in honeydew from Poland to understand the variability of species in honeydew originating from an area with a temperate climate. Our work indicates for the first time that honeydew from the temperate climate of Europe can be a promising source of new fructophilic lactic acid bacteria. To the best of our knowledge, the selected *Lb. plantarum* FPL strain is the first strain described as *Lactobacillus plantarum* with fructophilic behavior. The presence of *Lb. plantarum* in honeydew must have an impact on the ecosystem of aphids, bees, or ants. It can be concluded that *Lb. plantarum* FPL is a syntrophic bacterium inhabiting the gastrointestinal tract of *Coccus hesperidum* L. and taking part in sugar metabolism.

## Electronic supplementary material

ESM 1 (DOCX 1323 kb)

## Abbreviations

LAB: Lactic acid bacteria
FLAB: Fructophilic lactic acid bacteria
MALDI-TOF: Matrix-assisted laser desorption/ionization-time of flight mass spectrometry
FYP: Fructose yeast peptone medium
GYP: Glucose yeast peptone medium
HPLC: High-performance liquid chromatography
