# Supplementary material for: Isolation and characterization of a new fructophilic Lactobacillus plantarum FPL strain from honeydew
Source: Ann Microbiol. 2018 Jun 1;68(7):459–70. doi: 10.1007/s13213-018-1350-2 (PMC6008367; doi:10.1007/s13213-018-1350-2)
Supplement: Supplementary file 1 — (DOCX 1323 kb) [file 13213_2018_1350_MOESM1_ESM.docx]

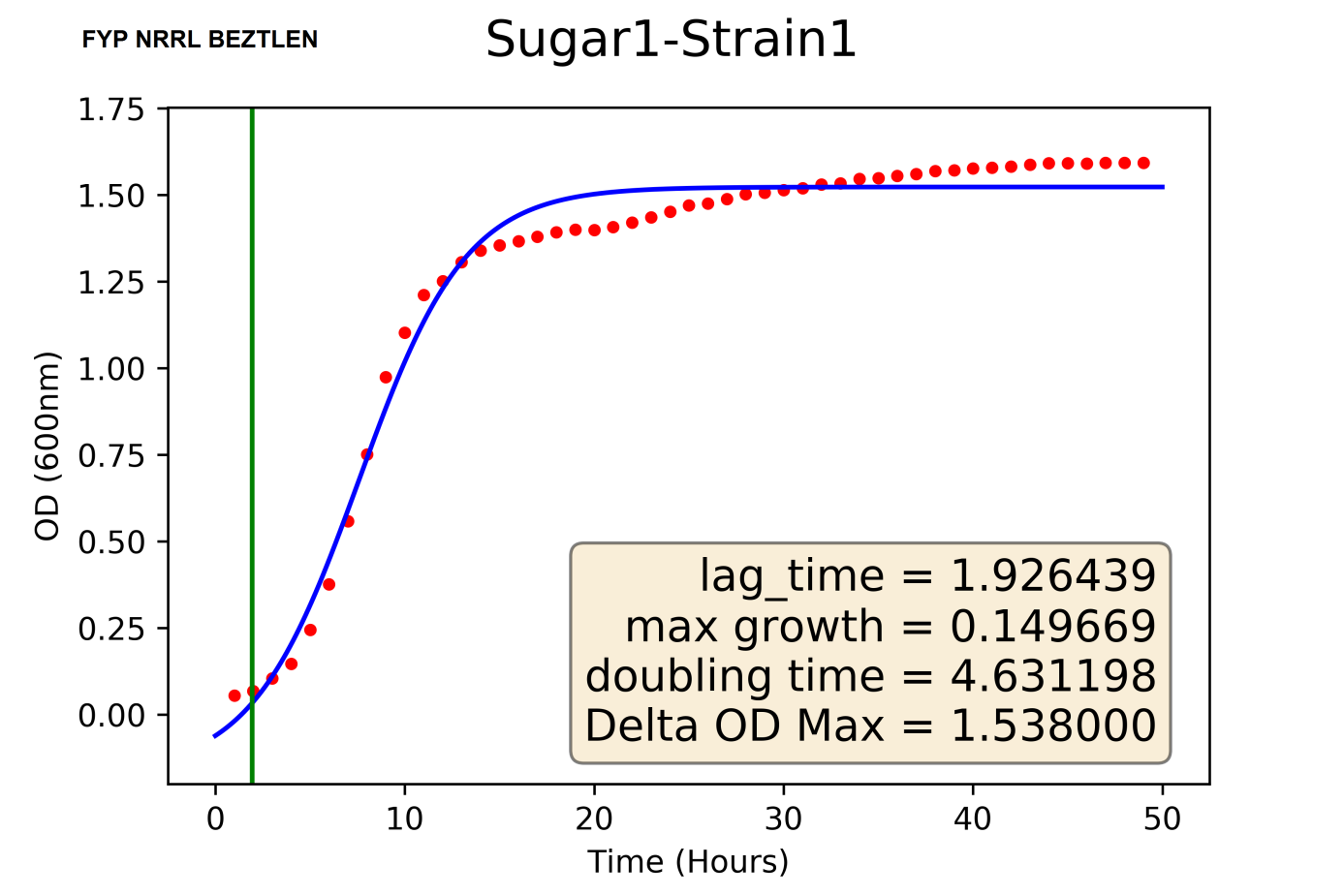


**Fig. 1** FYP medium*, Lb. plantarum* NRRL, anaerobic conditions


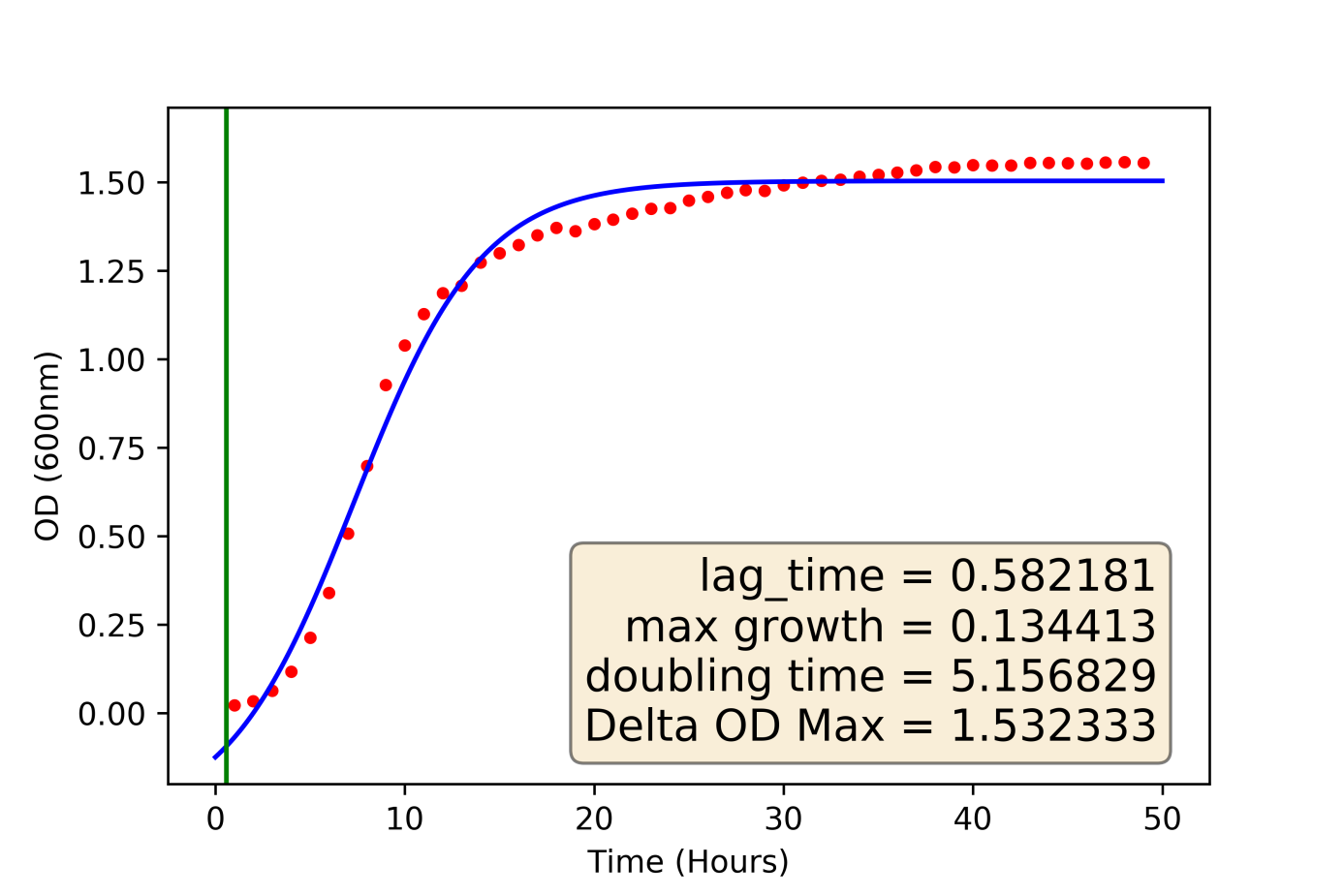


**Fig. 2** FYP medium*, Lb. plantarum* NRRL, aerobic conditions


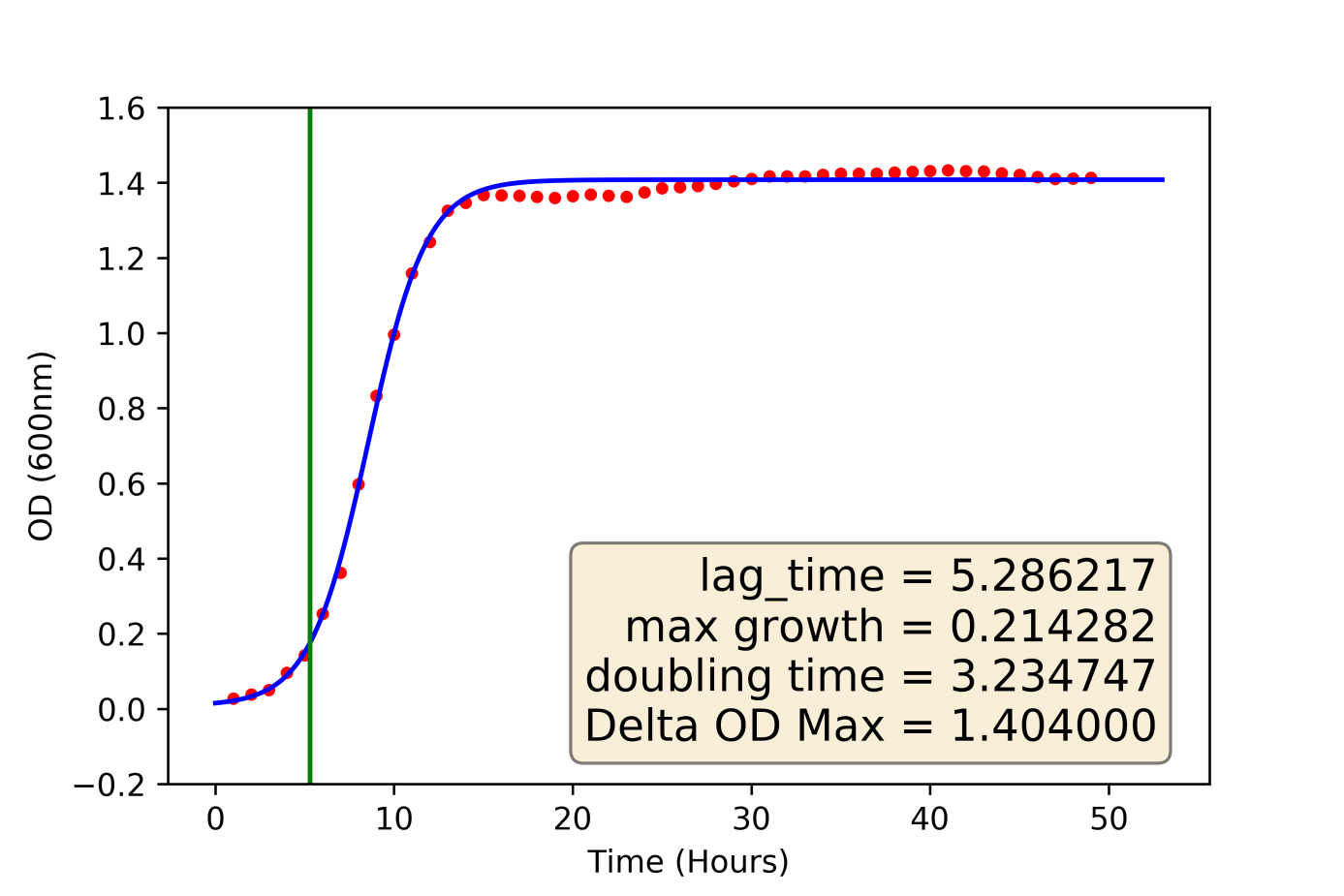


**Fig. 3** FYP medium, *Lb. plantarum* FPL, aerobic conditions

**
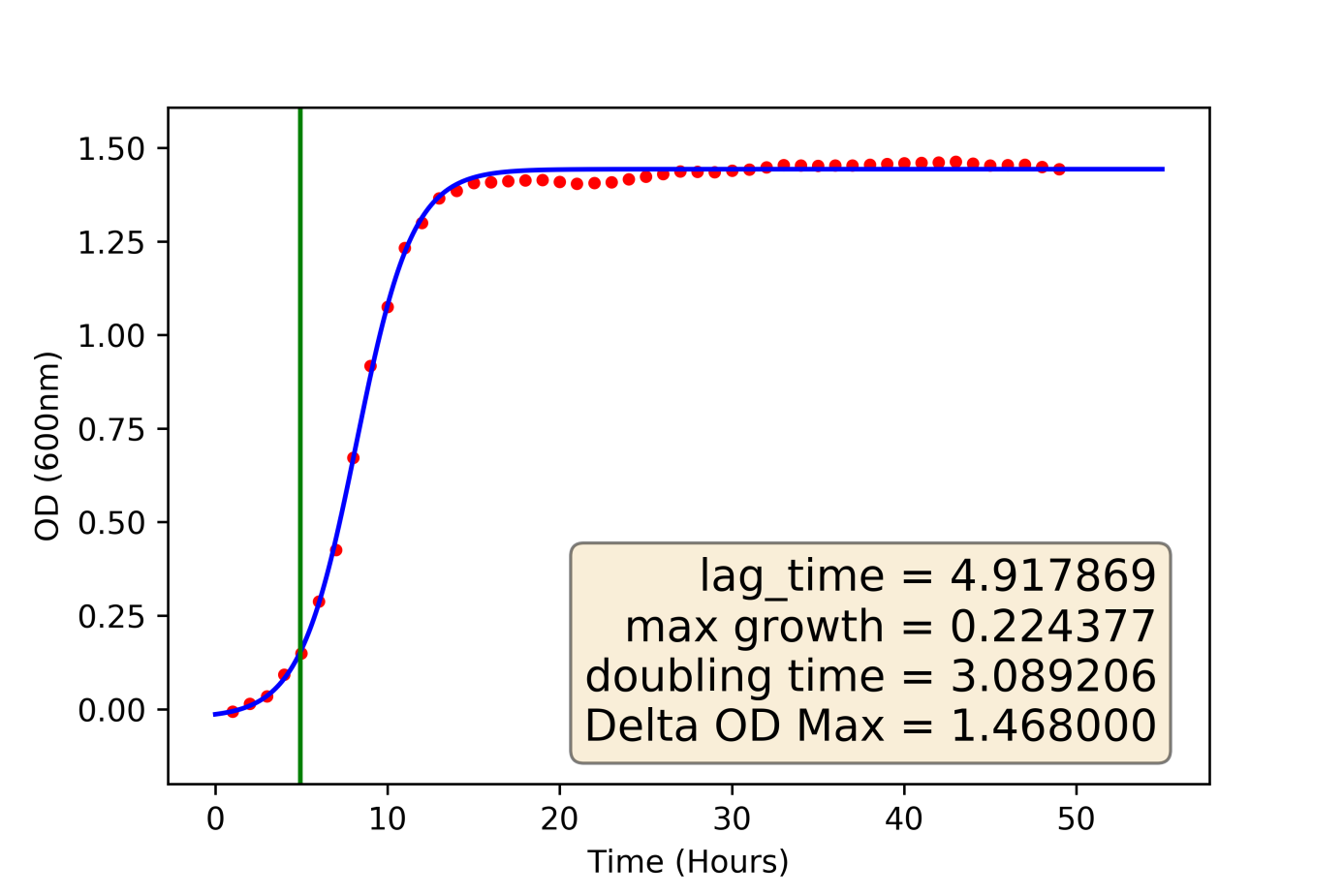
**

**Fig. 4** FYP medium, *Lb. plantarum* FPL, anaerobic conditions


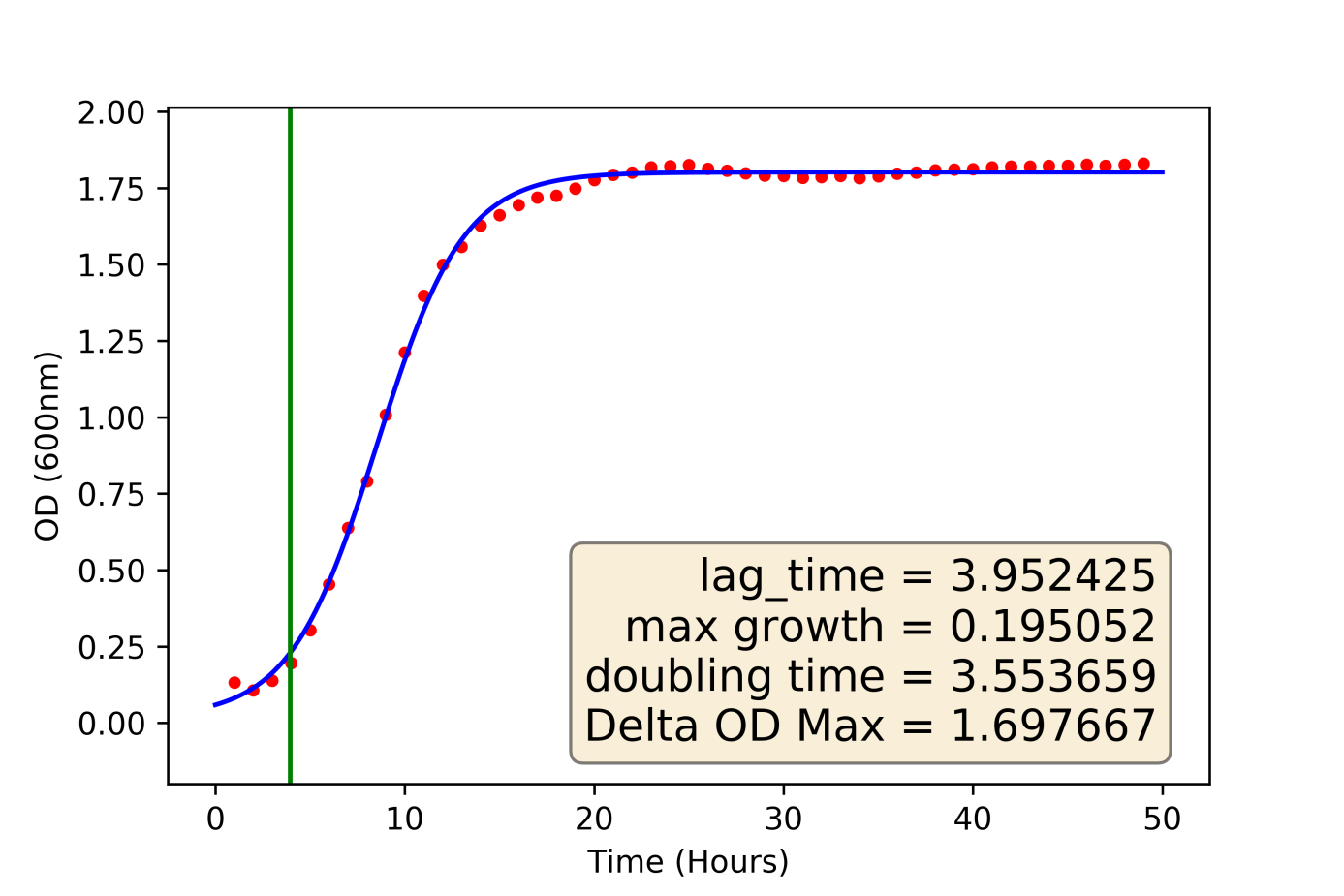


**Fig. 5** GYP medium, *Lb. plantarum* NRRL anaerobic conditions


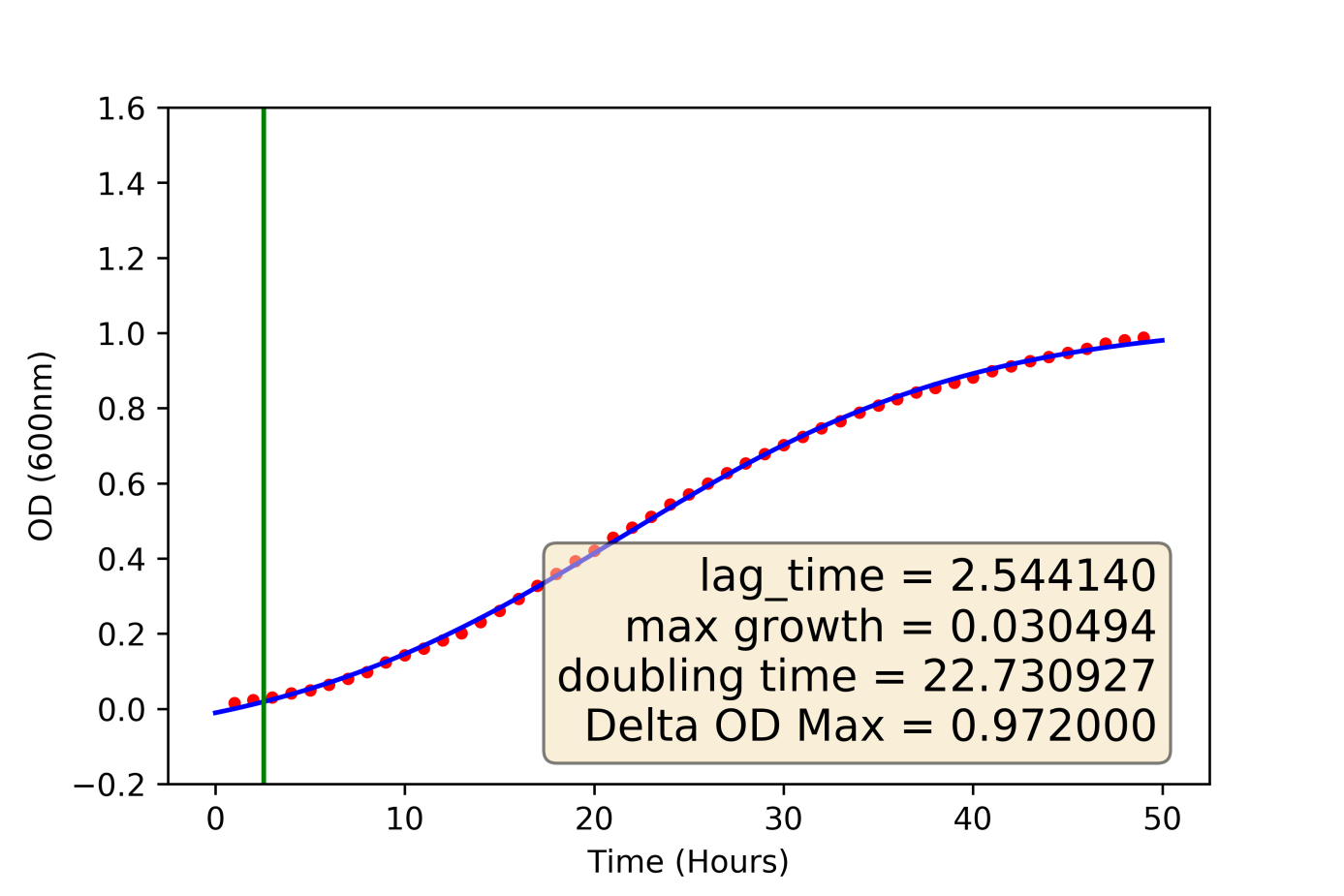
**Fig. 6** GYP-P medium*, Lb. plantarum* NRRL aerobic conditions


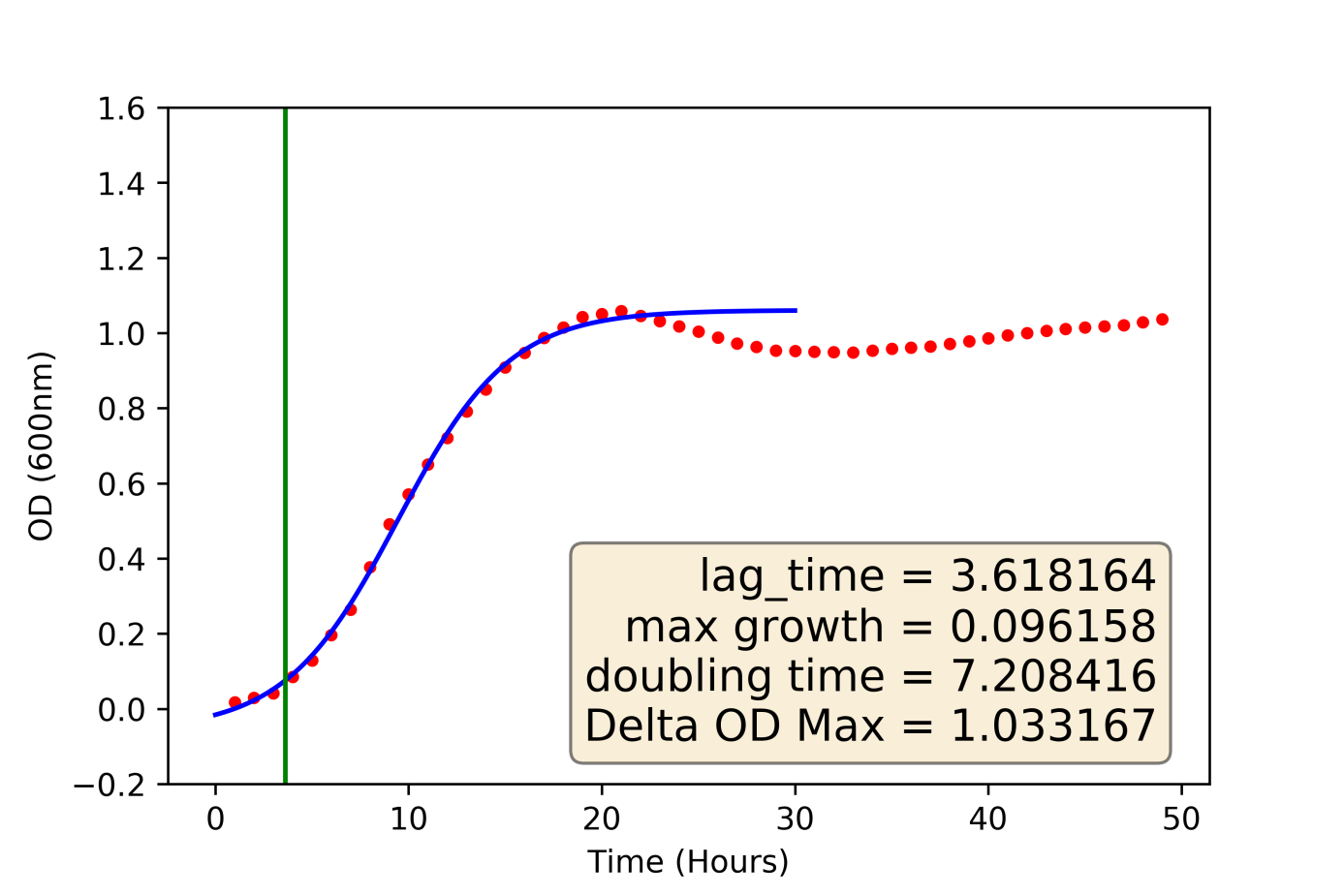


**Fig. 7** GYP medium, *Lb. plantarum* FPL, aerobic conditions


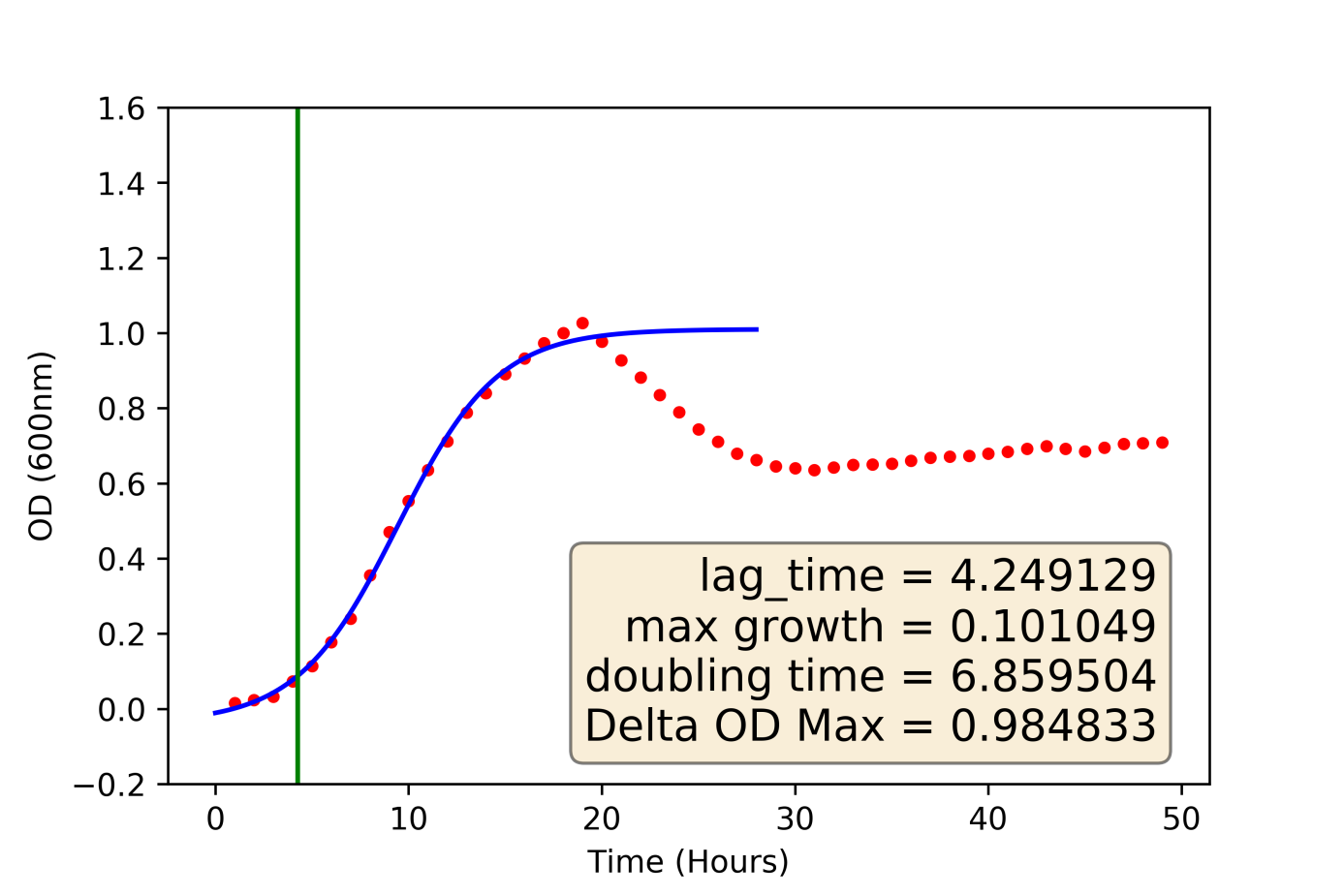


**Fig. 8** GYP medium, *Lb. plantarum* FPL, anaerobic conditions


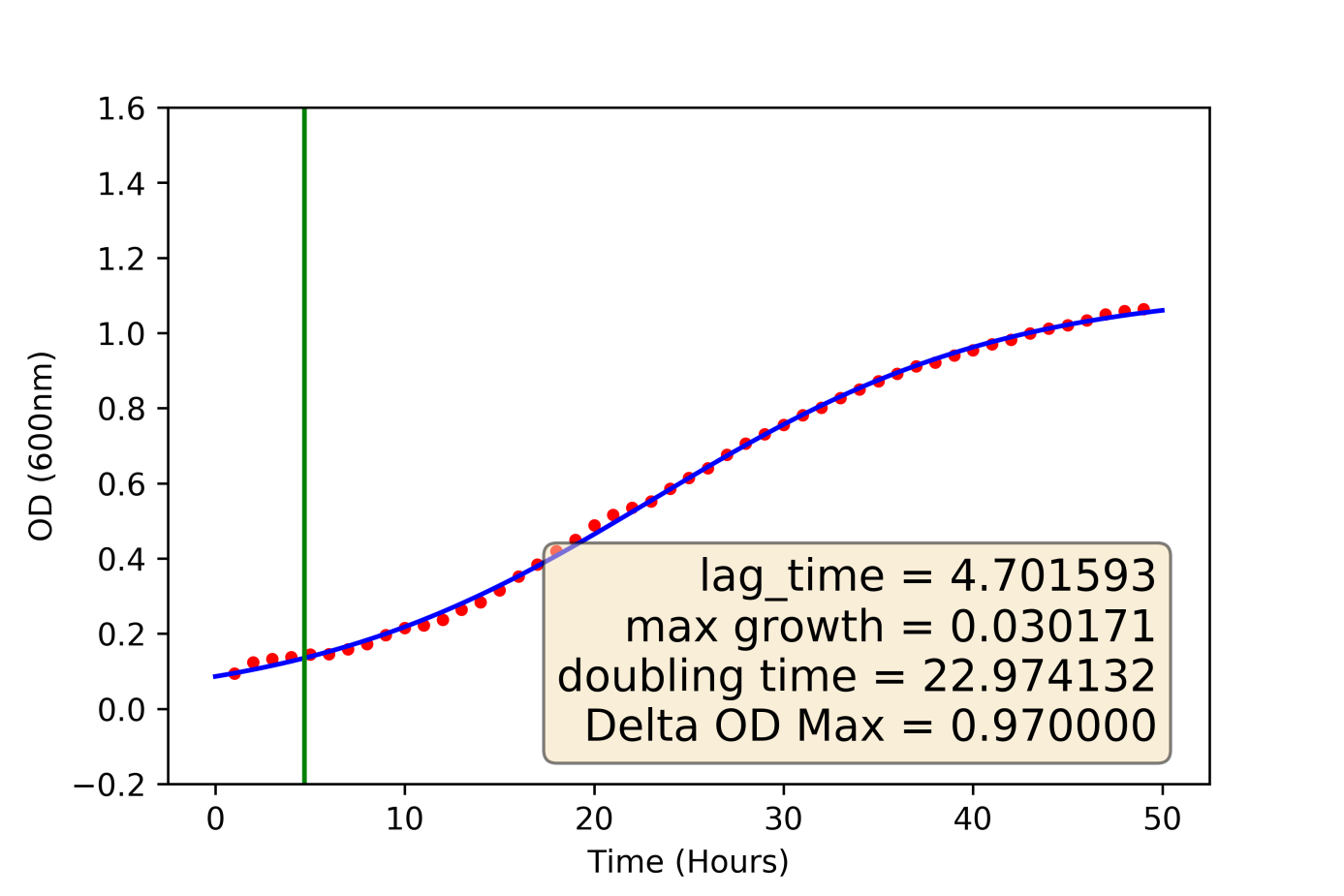


**Fig. 9** GYP-P medium, *Lb. plantarum* NRRL, anaerobic conditions


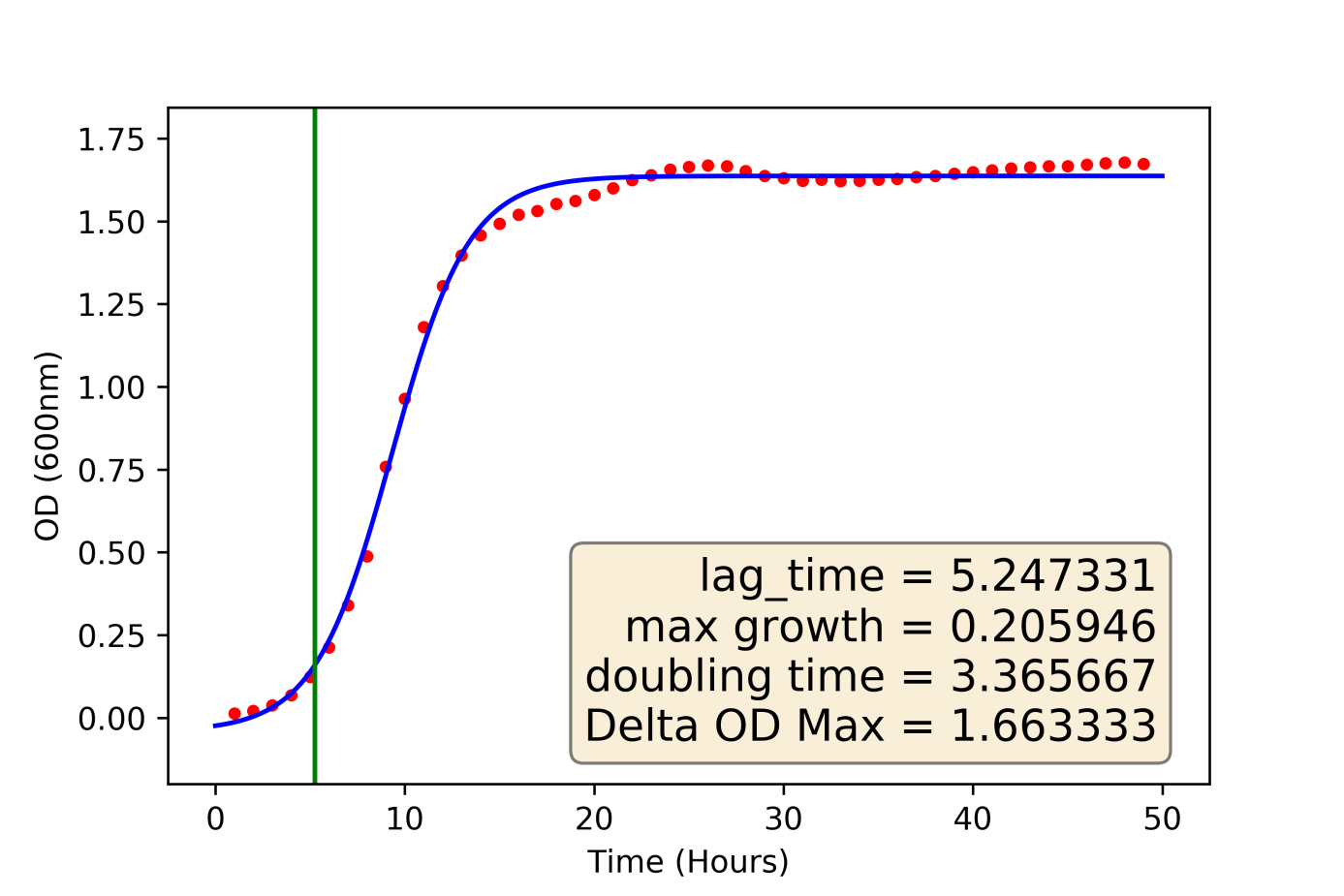


**Fig. 10** GYP medium *Lb. plantarum* NRRL, aerobic conditions


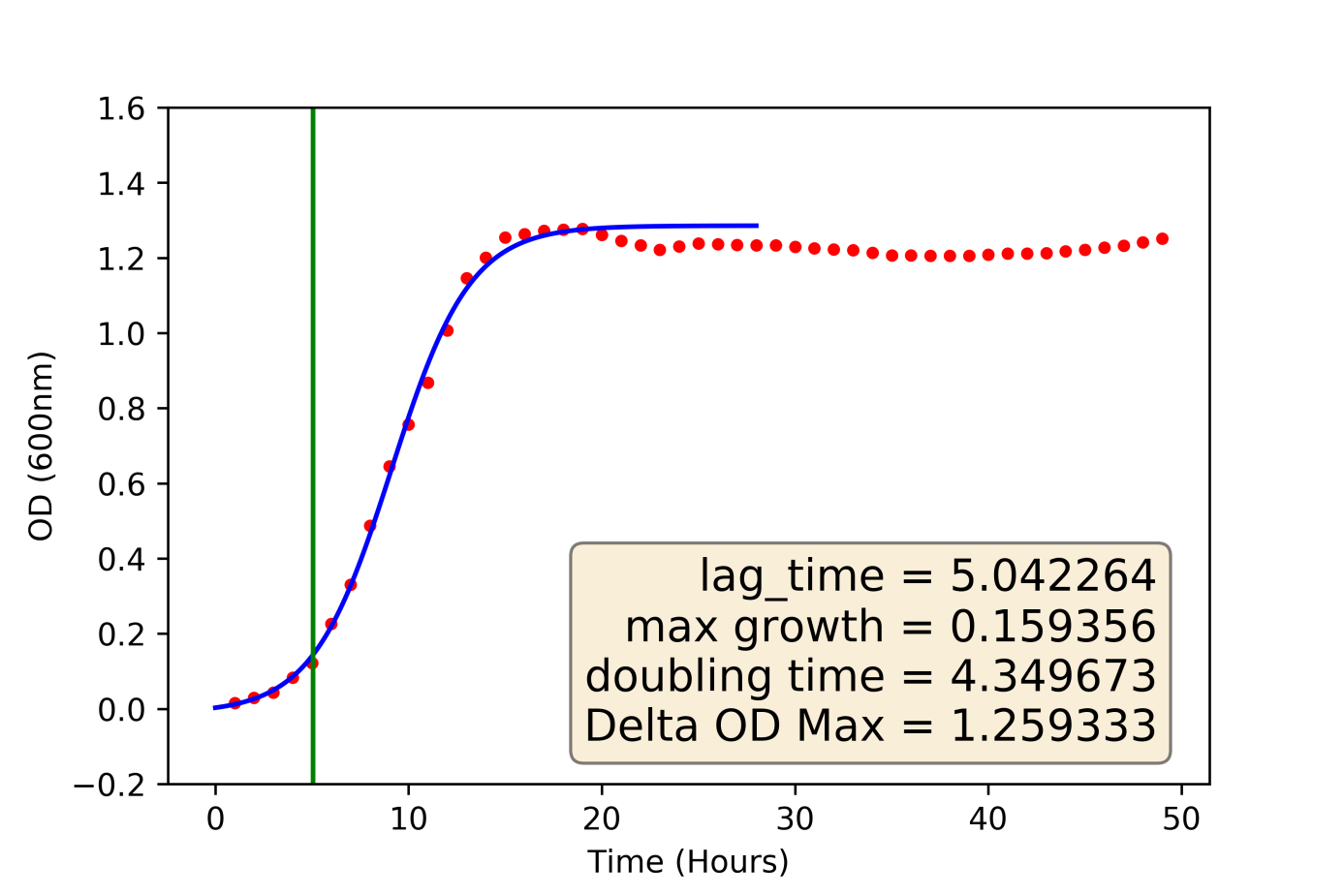


**Fig. 11** GYP-P, *Lb. plantarum* FPL, aerobic conditions


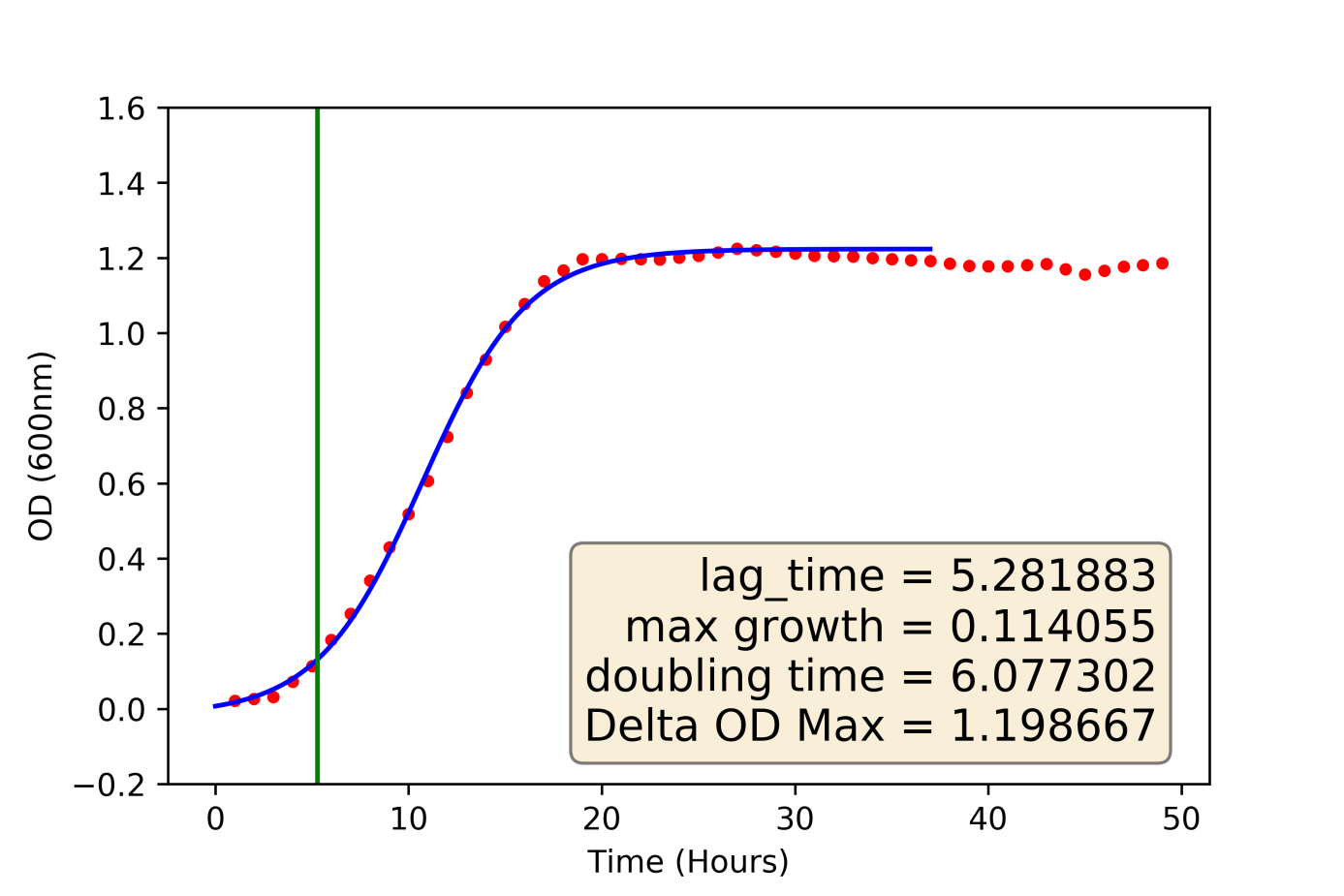


**Fig. 12** GYP-P, *Lb. plantarum* FPL, anaerobic conditions
